# Temperature profiles of field-aged photovoltaic modules affected by optical degradation

**DOI:** 10.1016/j.heliyon.2023.e19566

**Published:** 2023-08-28

**Authors:** Oscar Kwame Segbefia

**Affiliations:** Department of Engineering Sciences, University of Agder, 4879 Grimstad, Norway

**Keywords:** Optical degradation, Resistivity, Temperature coefficient, Thermal imaging, Co-defect

## Abstract

Moisture ingress into PV module in the presence of ultraviolet radiation, high temperature, and other environmental stressors can affect the optical integrity of the PV module. Optical degradation can take the form of delamination, discolouration of encapsulant, metal grids corrosion, and trapped moisture or chemical species. This can influence the photon absorption and current transport properties in the PV module bulk, which can affect the module operating temperature. In the present work, the relationship between optical degradation and temperature sensitivity of 20-year-old multicrystalline silicon field-aged PV modules have been investigated. The selected PV modules were characterized using visual inspection, current-voltage (I–V) characterization, temperature coefficients profiling, current resistivity profiling, infrared (IR) thermal, ultraviolet fluorescence (UV–F), and electroluminescence (EL) imaging. PV modules affected by optical degradation show weak fluorescence and luminescence signal intensities. The average difference in cell temperature (*ΔT*) between the warmest and coldest cell for the PV modules investigated was found to be around 10 ± 2 °C and the average power degradation rate was approximately 0.8% per year. The underlying factor for the observed degradation is attributed to the degradation in the temperature coefficients of open circuit voltage (*β*_*Voc*_) and maximum power point voltage (*β*_*Vmpp*_). The average temperature coefficient of efficiency (*β*_*ηm*_) of the modules was found to be around −0.5%/°C. Finally, a temperature dependent resistivity method for extracting temperature coefficients from IR thermal data of PV modules has been proposed.

## 1 Introduction

One of the most critical characteristics of good photovoltaic (PV) front encapsulation materials is optimum optical transmission efficiency [1,2]. However, in the field, PV modules are exposed to a variety of environmental stressors: high temperature, humidity, ultraviolet radiation, wind and snow loads, and soiling [[3], [4], [5]]. In the presence of these environmental stressors, moisture can diffuse into the bulk of the solar panel through the edge, back of the panel, and/or voids (e.g., cracks) created in the panel [4,5]. Moisture and moisture induced degradation (MID) products in the PV module initiate several degradation processes in the ethylene vinyl acetate (EVA), which is the most popular PV module front encapsulation material [4]. MID products include oxides, acetates, carbonates of metal grids e.g., silver, lead, tin, copper, and aluminum [5]. For instance, MID of the EVA encapsulation produces acetic acid, and possibly, silver acetate [6]. The MID species can lead to delamination and discolouration of encapsulants, snail trails, potential induced degradation (PID), loss of adhesion, corrosion of metal grids and other components of the PV module [1,6]. These degradation mechanisms have also been found to serve as a precursor for other degradation mechanisms in PV plants [1,4,7,8].

**Fig. 1:**
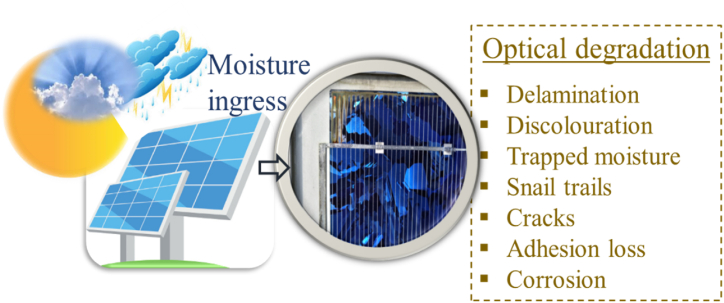
PV module in the field. Under environmental stressors e.g., high humidity, temperature, and UV radiation, moisture can enter the PV module. Moisture ingress can lead to optical degradation [5].

More importantly, the majority of these reliability issues affect the optical efficiency of the front encapsulant, and hence, constitute optical degradation [3]. For instance, metal grids corrosion and PID can lead to optical degradation and vice versa [9]. The route to optical degradation of PV modules due to moisture ingress is illustrated in Fig. 1. Over time, the issue of optical degradation becomes more pronounced and can in the most severe cases constitute more than 50% degradation in the rated power output of the PV module [7]. The loss of power output is due to increased optical reflection due to “light decoupling” with reduced photon absorption in the active PV material [1,7,10]. This has dire implications for PV module efficiency and costs for operating PV plants over their guaranteed lifetime. The optical transparency can be quantified by the “yellowness index”. According to the International Standards Organization [11], “yellowness index” is a measure of the deviation in polymer hue from colourless or whiteness toward yellow.

Pern et al. [10] observed ca. 50% reduction in the efficiency of PV modules as the encapsulant colour changed to dark brown. Rosillo and Alonso-García [12] reported up to 3% reduction in the maximum power (*P*_max_) of silicon crystalline PV modules due to high yellowness index. Dechthummarong et al. [3] investigated the relationship between encapsulant degradation and electrical insulation properties of field-aged single crystalline silicon PV modules that were deployed in Thailand. They observed that the modules with lower yellowness index possessed better electrical insulation properties. However, sometimes, the use of the “yellowness index” as a measure of optical degradation can be misleading [13]. For instance, de Oliveira et al. [13] observed up to 0.5%/year loss in power for ∼15 years field-aged PV modules with insignificant discolouration. In addition, degradation in power due to optical degradation also depends on other attendant defects and fault mechanisms within the module [[14], [15], [16], [17]]. It is therefore important to understand the characteristics of defects and fault mechanisms that lead to optical degradation of PV devices in time for the prevention of further deterioration and evolution of other failure mechanisms.

Several studies on detecting optical degradation in PV plants using visual inspection, infrared (IR) thermal imaging, electroluminescence (EL) imaging, and reflectance measurements have been carried out and documented [12,13]. Some reports also investigated the optical integrity of the front encapsulant and glass using fourier transform attenuated total reflectance (ATR-FTIR), differential scanning calorimetry (DSC), scanning electron microscopy (SEM) and energy dispersive X-ray spectroscopy (EDS), scanning auger electron microscopy (SAM), X-ray photoelectron spectroscopy (XPS), and secondary ion mass spectroscopy (SIMS) [12,13,18].

Hypothetically, variations in the module temperature coefficients can be traced to optical and/or electrical degradation [19]. According to earlier reports, optical degradation affects the electrical performance characteristics: especially the short circuit current (*I*_*sc*_) and the fill factor (*FF*) of the PV modules, which lead to subsequent power degradation [1,7]. Optical degradation influences the electronic charge transport properties in the PV module bulk [20,21]. In addition, optical degradation increases the number of UV absorption chromophoric species in the encapsulant [22,23]. These chromophores increase the UV absorption efficiency of the encapsulant with its attendant increased module operating temperature [24]. Moreover, the chromophores can also absorb/block visible light, hence, reduce the amount of useable photons reaching the active solar cell materials [7,25]. This leads to accumulation of current in the affected areas accompanied with high localized inhomogeneous cell temperatures known as hotspots in defective modules [14,18]. For ‘good’ modules, the temperature distribution is homogeneous [14].

**Fig. 2:**
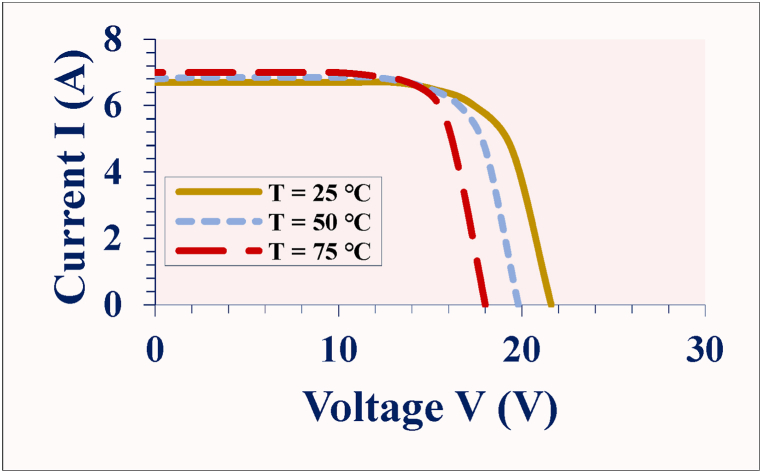
Effect of temperature on electrical characteristics of a normally operating PV module.

IR thermal imaging provides information on the temperature distribution over the PV module surface and the position of the defect or fault mode, hence, the defective cell or cells [18]. The temperature difference (*ΔT*) between the solar cell with the lowest temperature, *T*_*cL*_, and the solar cell with the highest temperature, *T*_*cH*_, can be an indicator of a specific defect or fault mechanism [14]. The nature of the hotspots depends on the characteristics of the defect and fault modes [2,14,18]. Also, the thermal profile of the module depends on the degree, defect density, and the areas affected by the hotspots [26]. Depending on the degree of optical degradation, the *ΔT* can be up to 6 °C or even higher [14]. High *ΔT* underpins mismatch losses [2,18,27]. Mismatch losses due to optical degradation can influence the overall PV module operating temperature (*T*_*m*_) [2,14,23]. High *T*_*m*_ affects the PV module efficiency (*η*_*m*_) and induces other degradation processes [4,14]. The best way to understand the effect of these degradation mechanisms on performance reliability is using field-aged PV modules which are exposed to multiple environmental stressors during operation. The effect of *T*_*m*_ on PV module performance is illustrated in Fig. 2.

In normal operation, the module voltage reduces significantly whilst the current increases but only slightly when temperature increases [28]. This affects the fill factor and efficiency of the module. Hence, *η*_*m*_ depends on *T*_*m*_ [19,29,30]. Moreover, the microscopic effect of temperature can accurately be traced to the temperature coefficients of the PV device [28,31]. The use of temperature coefficients for PV module fault diagnostics is non-destructive, fast, reliable, cost-effective, and can be done conveniently anytime. Studies on PV module temperature coefficients are well documented [19,28,29,32,33]. Dubey et al. [32] found that there is a good agreement between the temperature coefficients of different solar panel technologies measured in the field with the values obtained in the laboratory. Dupré et al. [28] reported that the physics of the temperature coefficients of solar cells depends on the loss mechanisms. Paudyal and Imenes [19] investigated the degradation of the temperature coefficients of solar panels installed in a Nordic climate using 8-years field data and found no degradation. Segbefia et al. [29] investigated the temperature sensitivities of 20-year-old field-aged multicrystalline silicon solar panels affected by microcracks. They observed ca. 1.2%/year in the efficiency of panels due to the degradation of the temperature coefficients. A review on the dependence of solar panels’ electrical performance on their temperature sensitivity is presented by Skoplaki and Palyvos [33].

The prospect of extracting the temperature coefficients from IR thermal data of PV modules is interesting, but non-existent. An additional analysis of temperature coefficients could make PV module fault diagnosis using IR thermal imaging more efficient, reliable, and cost-effective. To the best of our knowledge there is no report on characterizing PV module optical degradation using temperature coefficients profiling presently.

In the present work, field-aged multicrystalline silicon (mc-Si) PV modules affected by optical degradation are investigated using temperature profiling. The selected PV modules were characterized using visual inspection, current-voltage (I–V) characteristics, temperature coefficients profiling, infrared (IR) thermal, ultraviolent fluorescence (UV–F), and electroluminescence (EL) imaging. The temperature coefficients of maximum power (*P*_max_), open circuit voltage (*V*_*oc*_), short circuit current (*I*_*sc*_), fill factor (*FF*), and module efficiency (*η*_*m*_) were studied. In addition, the temperature coefficients of the maximum power point voltage (*V*_*mpp*_) and current (*I*_*mpp*_) were also examined. In Section 2, brief background information on the field-aged multicrystalline silicon (mc-Si) PV modules and the methods used for the investigation are presented. The results and the discussion follow in Section 3. A proposed temperature dependent resistivity technique for defects and faults diagnosis in PV modules is presented in Section 4.

## 2 Material and methods

**Fig. 3:**
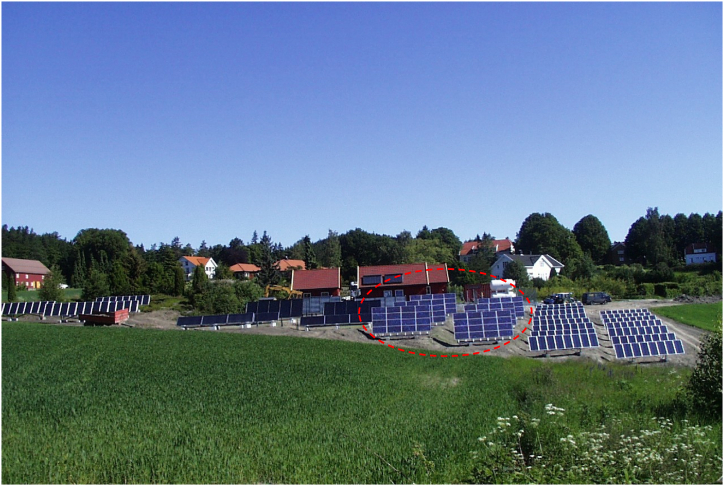
The Renewable Energy Park located at Dømmesmoen. The mc-Si PV modules under study are shown in a red circle.

In Summer of the year 2000, the Renewable Energy Park located at Dømmesmoen (58.3447° N, 8.5949° E) in Norway was commissioned as a resource center for research and education in renewable energy. The Park contained 96 NESTE NP100G12 mc-Si PV panels (in a red circle), as well as amorphous silicon (a-Si) panels (extreme right and left) and thermal collectors (immediate left) as shown in Fig. 3. The mc-Si panels were rated 100 W each [34]. However, in 2011, the park was decommissioned, and the PV panels were kept securely indoors for research purposes. At the time of decommissioning, the maximum power of the mc-Si panels had dropped to ca. 90% [35]. In earlier reports on the field-aged modules, about 90% of the mc-Si panels have been affected by optical degradation [5,8].

In the present study, 3 of these field-aged PV modules (A, B, and C) which have been affected by optical degradation have been selected for investigation. The electrical characteristics of the 3 selected modules and the manufacture's data sheet values are presented in Table 1. The information on the modules and solar cells has been presented earlier [5,36].

**Table 1:** Average electrical parameters of the 3 selected field-aged PV modules, normalized to STC.

PV module		*P*_max_ (W)	*V*_*oc*_ (V)	*I*_*sc*_ (A)	*V*_*mpp*_ (V)	*I*_*mpp*_ (A)	*FF* (%)	*η* (%)
2000 (Data sheet)		100	21.6	6.7	16.7	6.0	70	13.0
2011 (Average)		90.2	21.5	6.2	16.1	5.1	68	12.0
**2021**	**A**	82.2	20.9	6.0	15.6	5.3	66	10.7
	**B**	84.1	20.9	6.0	15.9	5.3	67	10.9
	**C**	82.1	20.7	5.9	15.5	5.3	67	10.7

### 2.1 Visual inspection

The field-aged PV modules were taken through a thorough visual inspection program in a clear sky outdoor environment. In addition, high-resolution photographs of each PV module were taken in a dark room under well-controlled light exposure. The visual inspection in the dark room reduced the undesired glare or gloss effect of the front glass. This made it possible to identify some details of the defects which are not easily seen with the traditional visual inspection technique alone. This ensures a comprehensive cataloguing of all visible defects and fault modes. The International Electrotechnical Commission: IEC 61215: 2016 standard was duly adhered to in collecting and reporting the data from the visual inspection of the PV modules.

### 2.2 I–V measurements

The field-aged PV modules were each taken through current-voltage (I–V) curve electrical performance measurements using a handheld I–V 500w I–V Curve Tracer, following the procedure according to IEC 60891 and the IEC 60904- 1 series. These measurements provided information on the *P*_max_, *V*_*oc*_*, V*_*mpp*_, *I*_*sc*_*, I*_*mpp*_, *FF*, in-plane irradiance (*G*_*I*_), and temperature characteristics of each module at Standard Test Conditions (STC). STC specifies cell temperature of 25 °C, an irradiance of 1000 W/m^2^ and air mass 1.5 (AM1. 5) spectrum for commercial PV modules. Measurements were done under in-plane irradiance conditions (970–1130 W/m^2^), and the I–V tracer used converted all measurements to STC automatically. This means the operating conditions were optimally resolved by the device to minimize errors in measuring and recording data. The difference in each electrical parameter, *x* (*Δx*), was computed as the difference between data sheet values and measured values in the year 2021.

### 2.3 Temperature coefficient profiling

To measure the temperature coefficients, the PV modules were taken out to the outdoor measuring rack one after the other from a storeroom where the modules were kept at room temperature. The PV module and the reference device were then covered with a shade (cardboard), and the measurement initiated immediately after the shade was removed. Module temperature was measured with an PT300 N probe (PT1000) attached to the backside of the PV module. The measurements on all modules were done on the same day between 12.30 and 14.30 h on a clear sky day and at wind speed less than 2 ms^−1^. In all cases, the PV modules were sun soaked for at least 30 min before the measurements were taken to allow sufficient time for the modules to reach thermal equilibrium. The IEC 60891-4 standard was followed during the investigation. However, according to this standard, the temperature range of the data values should be at least 30 °C. This seems practically challenging on the investigation site. The electrical parameters (*P*_max_, *V*_*oc*_, *I*_*sc*_, *FF, η*_*m*_*, V*_*mpp*_*, I*_*mpp*_) were plotted as functions of PV module's temperature (*T*_*m*_) and a least-squares-fit curve through each set of data was constructed. The regression equation for such a relation can be represented as *y = mx + c*, where *m* and *c* are the slope and the intercept, respectively. The relative temperature coefficient of parameter *X* (*β*_*x*_) in %/°C was calculated by dividing the slope (*m*) of parameter *X* by the intercept (*c*) of parameter *X*. That is, *β*_*x*_ *= m/c*. Details of this measurement procedure was reported earlier [27,36].

### 2.4 Ultraviolet fluorescence (UV–F) imaging

In the detection of defects and fault modes, including cracks and optical degradation, the UV-F imaging is one of the most suitable tools [37,38]. PV components, especially polymeric materials degrade into fluorescent species when exposed to environmental stressors and chemical species. In the presence of ingressed moisture or other gaseous species such as oxygen, the fluorescent degraded species undergo metamorphoses to nonfluorescent species via photobleaching or photoquenching [37]. These nonfluorescent species form photobleaching marks around and within the defective areas in the module and show darker traces when exposed to UV-F [38]. UV-F images of the field-aged PV modules were taken in a dark room using a TROTEC® LED UV TorchLight 15F (λ ≈ 360 nm) together with a Wolf eyes FD45 spectrum filter. The IEA prescribed procedure was followed in the investigation [16,39].

### 2.5 Electroluminescence (EL) imaging

EL imaging works on the principle that when a PV module is forward biased, the solar cells glow in the near-infrared (NIR) region; peaking around 1150 nm for silicon cells [26]. This signal could be captured with an infrared camera, and the degradation state of the cells and the balance of system (BoS) materials could be extracted from the EL images. EL imaging is interesting especially for quantifying resistive losses in old PV modules affected by cracks and severed metal grids. Moreover, the technique could also be very useful in detecting encapsulant degradation [40]. Degraded encapsulant reduces the luminescence signal that gets through to the detection camera. The PV modules were taken through the EL characterization in a dark room using the BrightSpot EL Test Kit. The kit comprises a 24 megapixels modified DSLR (digital single-lens reflex) Nikon D5600 camera, power supply set, and computer with data acquisition and post processing software. The image acquisition and processing were done according to the IEA procedure [16,26] and the IEC TS 60904-13 standard. The EL characterization of the sampled PV modules was done indoors using 10% and 100% of the *I*_*sc*_ 5 min after the current was initiated.

### 2.6 Infrared (IR) thermal imaging

**Fig. 4:**
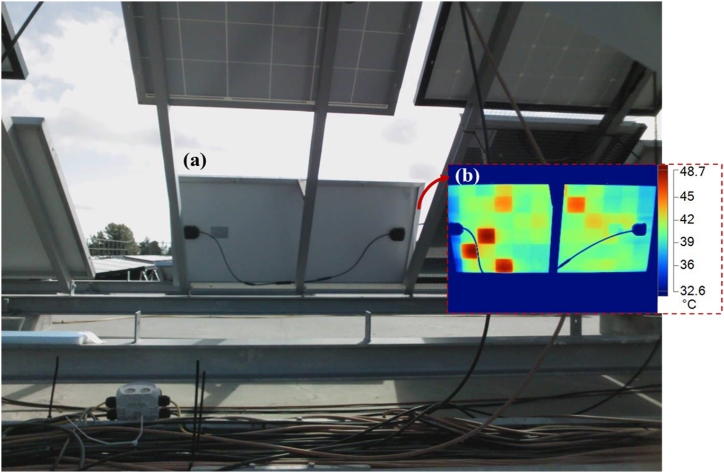
(a) Visual image and (b) IR thermal image (insert) of a PV module from the backside during IR thermal imaging. The metal support (behind the PV module) and the cables show corresponding blue traces in the IR thermal image.

When a PV module is forward biased, current accumulates on cell areas affected by defects and fault modes. This leads to localized hotspots or joule heating which raises the PV module's temperature and increases the intensity of the emitted IR radiation. This signal could be captured with an infrared thermal camera, and the thermal state of the cells could be extracted from the IR thermal images. The PV modules were taken through infrared measurements using the Fluke Ti400 Infrared Camera (measuring in the long-wave IR band: 650–1400 nm) by following the IEA prescribed procedure [26] and IEC TS 62446-3 standard. Measurements were done under clear sky outdoor conditions. The experimental set up for the IR thermal imaging is illustrated in Fig. 4. Fig. 4a and b shows the visual image and IR thermal image (insert) of a PV module from the backside during IR thermal imaging, respectively. IR thermal images were acquired after soaking the PV modules in the sun for at least 15 min.

## 3 Results and discussion

### 3.1 Visual inspection

**Fig. 5:**
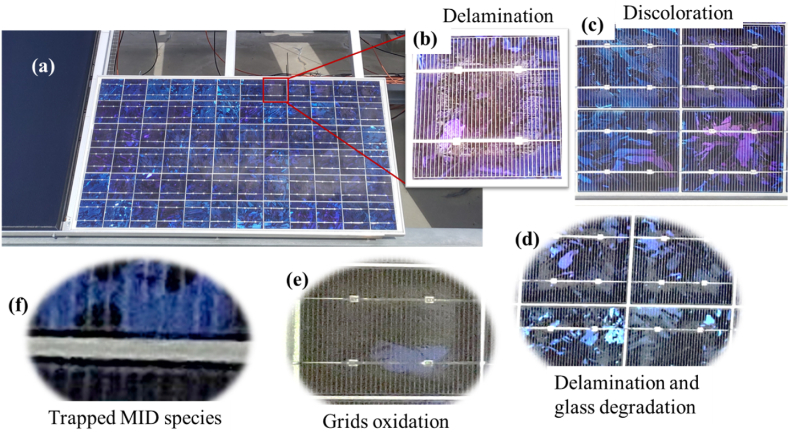
Defects and fault modes of the field-aged PV modules affected by optical degradation. Photographic images showing (a) one of the PV modules, (b) a solar cell affected by delamination around the solar cell edges, (c) discolouration of the front encapsulant, (d) delamination around the solar cell edges and front glass degradation, (e) metal grids oxidation at the solder joints, (f) trapped moisture induced degradation (MID) species around the solar cell edges.

A change in the colour of encapsulant towards yellow and dark brown indicates optical degradation [1,7,12]. However, the effect of optical degradation could precede the change in the colour of the encapsulant [13]. Yet, the most established technique for detecting optical degradation is the visual inspection [1,16]. Fig. 5 shows some of the results from the visual inspection. Fig. 5a is a photographic image of one of the field aged PV acquired under clear sky conditions. The field-aged PV modules show cells with delamination at the cell edges (Fig. 5b), discolouration of the EVA encapsulant (Fig. 5c), front glass degradation (Fig. 5d), oxidation of metal grids at the solder joints (Fig. 5e), and trapped moisture induced degradation (MID) species around the solar cell edges (Fig. 5f), as reported earlier [6]. Also, some of the modules have loose aluminium (Al) frames and microcracks which served as a conduit for moisture ingress, as reported earlier [5]. These defect and fault mechanisms are the underlying factor for the degradation mechanisms observed in the field-aged PV modules, as reported in other studies [4,9].

According to Tsanakas et al. [14] optical degradation includes encapsulant discolouration, delamination, glass breakage, and trapped moisture or bubbles. However, in this work, none of the field-aged PV modules under investigation have visible broken glass. The denominator for the observed optical degradation is moisture ingress considering the climatic conditions of the installation site [4,5,17].

### 3.2 Ultraviolet fluorescence (UV–F) characteristics

**Fig. 6:**
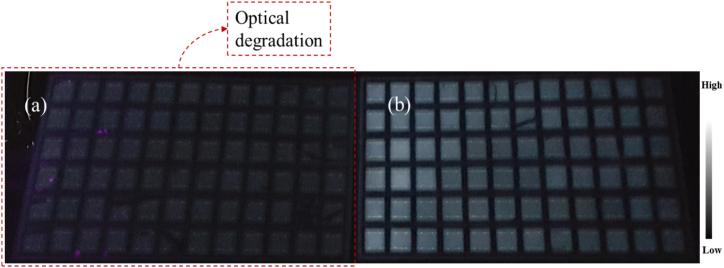
Ultraviolet fluorescence (UV–F) images of two field-aged PV modules showing the two classes of modules: (a) optical degradation (Class I) and (b) ‘good’ front encapsulant (Class II). The 3 field-aged PV modules under this study belong to Class I.

Fig. 6 shows UV-F images of the two classes of the PV modules investigated as per their fluorescence intensity. Based on the fluorescence signal intensity from the front encapsulation of the field-aged PV modules, the PV modules were classified into 2 classes, as reported earlier by the same authors [5]. For this purpose, the focus is on the response of the front encapsulant to UV-F light in order to determine which modules have been affected by optical degradation and which are not. Hence, we ignore other defects (e.g., cracks) that appear in the UV-F images of the PV modules. Class I modules (Fig. 6a) show very weak fluorescence intensity, and their surfaces appear uniformly darker. Class II modules (Fig. 6b) show relatively strong fluorescence intensity and appear brighter under the UV light. Class I PV modules constitute more than 90% of the field-aged PV modules. This indicates that the front encapsulation of the class of PV modules in Fig. 6b are in relatively better condition. It is noteworthy that both classes of PV modules do not differ significantly in terms of physical appearance and power output [5]. This suggests that the power degradation in the PV modules is not due to optical degradation alone.

### 3.3 Electroluminescence images

**Fig. 7:**
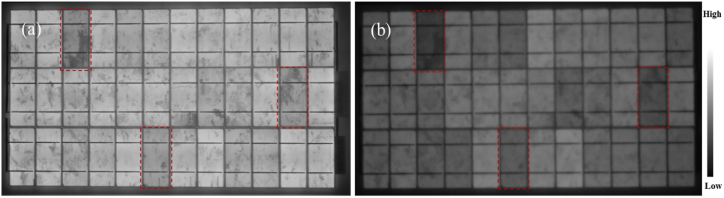
EL images of a new silicon mini-PV module under (a) 100% of *I*_*sc*_ and (b) 10% of *I*_*sc*_ conditions. Corresponding cell degradation areas are marked in red.

In a PV module with ‘good’ encapsulation, a strong luminescence signal intensity is recorded by the near IR (NIR) camera. That is, EL intensity can be an indicator of PV module degradation. Fig. 7 shows a ‘good’ PV module imaged under 100% of *I*_*sc*_ and 10% of *I*_*sc*_ forward bias conditions, respectively. A close inspection of both images shows that the front encapsulant of this PV module is in good condition, even though the luminescence signal intensity in Fig. 7b is weaker. The strong luminescence signal intensity for good encapsulant in Fig. 7 is due to the fact that good encapsulants have high optical transparency, hence, majority of the luminescence signal passed through the encapsulant and were detected by the NIR camera. The relatively weak luminescence signal recorded in Fig. 7b is due to reduced forward bias current: 10% of *I*_*sc*_ with high voltage [14]. The defects that are highlighted in Fig. 7a; the EL image acquired under *I*_*sc*_ forward bias conditions are also highlighted in Fig. 7b; the EL image acquired under 10% *I*_*sc*_ forward bias conditions. These defects (marked in red) are related to cell degradation during production, transportation, and handling since this module is yet to be installed outdoors. In addition, under 10% *I*_*sc*_, more material (cell) degradation issues are highlighted, as observed elsewhere [26,41]. On the other hand, in PV modules affected by encapsulant degradation, the intensity and profiles of the luminescence signal could be different [41]. This is true for field-aged PV modules which have undergone significant degradation due to exposure to several environmental stressors.

**Fig. 8:**
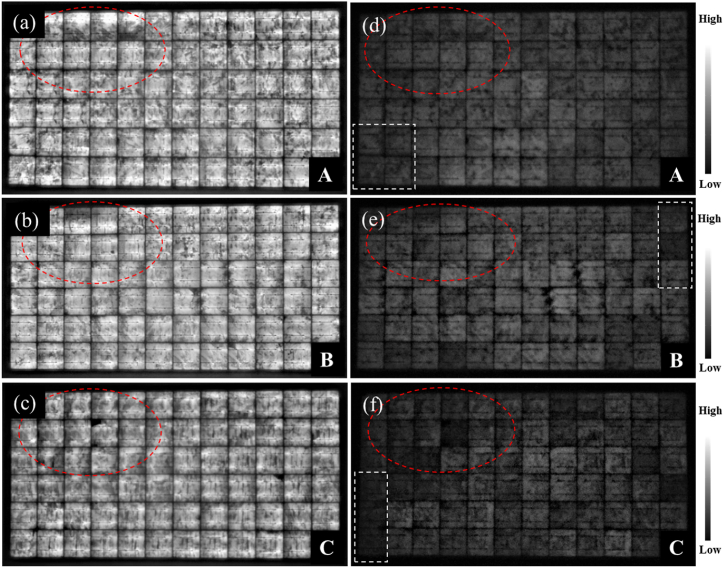
EL images of the 3 field-aged PV modules under (a)–(c) 100% of *I*_*sc*_ and (d)–(f) 10% of *I*_*sc*_ forward bias conditions. Corresponding defect areas in both EL images are circled in red. Areas marked in white are the corresponding areas marked in the IR thermal images in Fig. 9.

The EL images of the 3 field-aged PV modules acquired under 100% *I*_*sc*_ (Fig. 8a–c) and 10% *I*_*sc*_ (Fig. 8d–f) are shown in Fig. 8. PV modules affected by optical degradation show randomly distributed small darker spots all over the PV modules in the EL images acquired under 100% *I*_*sc*_. In addition, localized hotspots along the busbars are also distributed randomly over the PV modules. Except for the cells affected by cracks (Fig. 8a–c), the darker cells in the EL images acquired under 100% *I*_*sc*_ do not clearly correspond with the darker cells in the EL images acquired under 10% of *I*_*sc*_ conditions.

According to Sinha et al. [18], the observed darker cells in EL images is due to resistance losses emanating from optical degradation and electrical mismatch. The extra darker cells observed in the EL images acquired under 10% *I*_*sc*_ indicate cell degradation [26]. The dark spots indicate areas of cell cracks, corrosion, delamination, and discolouration of encapsulants and other material degradation e.g., solder bond degradation [42]. Accumulation of current at the shunts of defect areas gives rise to the darker spots [41]. The weaker luminescence signal intensity recorded in Fig. 8 for the field-aged PV modules is due to the fact that the encapsulants of these modules have undergone optical degradation, hence, blocking the greater portion of the luminescence signal from the cells.

The degradation of the solar cells could also be the reason for the observed weaker EL intensity in Fig. 8. However, defective cells e.g., cells affected by cracks show characteristic darker patterns, refer to Fig. 8d–f and Fig. 7. Moreover, Fig. 7, Fig. 8 were acquired under the same forward bias *I*_*sc*_ conditions. However, the quality of the EL images in Fig. 7, Fig. 8 are totally different. This is as a result of the degradation states of the field-aged PV modules, see Fig. 8. The quality of the EL images in Fig. 8 is affected by the optical characteristics of the front encapsulation material and the solar cells due to degradation..

**Fig. 9:**
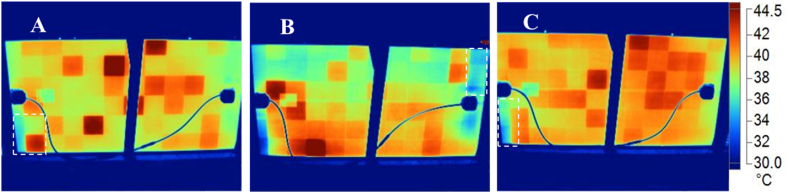
IR thermal images of PV Module A, B, and C under clear sky outdoor conditions. Corresponding defect areas in Fig. 8 are marked out in white.

### 3.4 IR thermal images

The infrared thermal images of the 3 field-aged PV modules are shown in Fig. 9. The solar cells with the highest temperature (*T*_*cH*_), which correspond to hotspots, spread randomly over the PV modules. A close comparison of the IR thermal images (Fig. 9) to the EL images (Fig. 8) suggests that the hotter cells correlate to the areas where the darker spots were observed in the EL images. The observed hotspot cells in the IR thermal images underline the observation of cell degradation as confirmed in the EL images [2,26]. Also, due to the uniform optical degradation of the modules, the observed randomly distributed hotspots are possible since optical degradation also leads to other degradation modes e.g., moisture ingress, PID, corrosion, solder bond degradation, etc. The bypass diodes of these modules were found to be in good condition [27]. An interesting observation is that some of the darker cells located at the edges of the PV modules in the EL images (Fig. 8) show lower cell temperatures in the IR thermal images (Fig. 9). A possible explanation for this observation is that these defect cells might be short circuited to the aluminium frames due to degradation mechanisms such as corrosion [26]. In an earlier microstructural investigation on these PV modules, the presence of MID species was observed, especially on the perimeter cells which show similar characteristics. These MID species lead to corrosion, optical degradation, PID, etc., and hence, shunting and other parasitic resistance loss mechanisms [6,8]. Changes in the *T*_*m*_ due to optical degradation leads to reduced bulk resistivity, hence, PID [16,43]. Moreover, optical degradation predisposes the module to moisture ingress which can lead to PID [5].

The shunts (due to optical degradation) created by the short circuits create alternative path for current [43]. That is, PV modules with optically degraded encapsulation can also serve as conduits for current leakages [44]. The marked defect cells in Fig. 9a–c might be reverse biased to the bypass diodes and therefore may possibly be operating under reverse bias conditions. Also, electrically isolated cells can show lower cell temperature (*T*_*c*_) since current cannot get to these areas easily [26]. Degradation of these cells into inactive areas around the perimeter of the PV modules might be due to moisture induced degradation of these cells. Around the perimeter of the PV modules, moisture ingress is likely, and can cause further degradation of the cells and its components [9]. In these situations, the defect cells may not show hotspots [26,38,39]. To further explore the relationship between optical degradation and module temperature sensitivity, data on the solar cells with the highest temperature (*T*_*cH*_) and lowest temperature (*T*_*cL*_) for each PV module over time was extracted and the difference in cells’ temperature (*ΔT*) was computed. That is,

(1) $$\Delta T=T_{cH}-T_{cL}$$

Table 2 shows the average values of *T*_*cH*_, *T*_*cL*_, and *ΔT* for the 3 field-aged PV modules. The average *ΔT* due to optical degradation was found to be ∼10 ± 2 °C.

**Table 2:** Difference in temperature (*ΔT*) of the solar cells with the highest temperature (*T*_*cH*_) and lowest temperature (*T*_*cL*_) for each field-aged PV module. Measurements were made under clear sky outdoor conditions.

PV module	Temperature (°C)		ΔT (°C)
	T_cH_	T_cL_	
**A**	47.7	36.2	11.5
**B**	52.3	41.7	10.6
**C**	44.2	33.7	10.5

Taking an error margin of ±2 °C into account, the observed *ΔT* values for the 3 field-aged PV modules agree with reported *ΔT* values that underlines optical degradation. In literature, *ΔT* due to optical degradation was found to be ca. ∼6.0 ± 2 °C or higher, based on the degree of the optical degradation and/or the presence of other defect/fault modes [2,14]. Optical degradation induces other failure mechanisms [4,18]. The presence of other defects in these PV modules (mainly due to moisture ingress) was reported by Segbefia et al. [5,6]. This observation was also reported elsewhere [18]. The location of majority of the darker cells in the EL images (Fig. 8) and warmer cells in the IR thermal images (Fig. 9) along the edges of the PV modules suggests that the presence of PID in these PV modules is likely [15,16,39]. According to Segbefia and Sætre [36], some of these modules have been affected by PID as well.

### 3.5 I–V characteristics

**Fig. 10:**
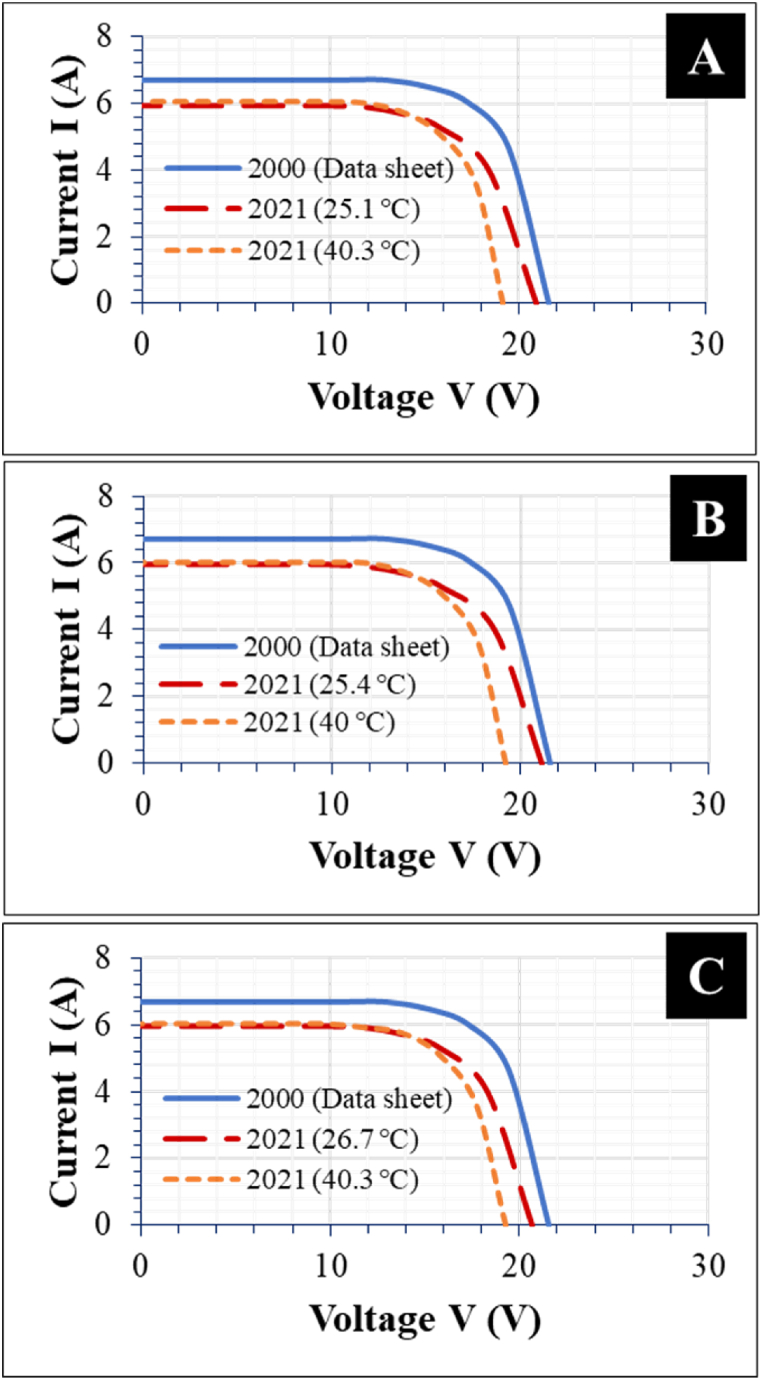
I–V profiles of PV Module A, B, and C. Permanent degradation in *I*_*sc*_ even when *T*_*m*_ increases indicate optical degradation.

Optical degradation influences photon absorption efficiency and current flow in the PV module bulk, especially in the areas affected by the optical defect. This constitutes current mismatch, which can cause local hotspots over the PV module [14]. High *T*_*m*_ affects *V*_*oc*_ and hence, fill factor (*FF*) and *P*_max_ [30]. The field-aged PV modules show a drop in the *P*_max_ due to decrease in optical efficiency. The efficiency of all the 3 modules decreased from 13% to less than 11%, refer to Table 1. This translates into a degradation rate of 0.8% per year in the module efficiency. Fig. 10 shows the I–V characteristics of the 3 PV modules at two different *T*_*m*_, normalized to STC.

All the 3 field-aged PV modules show similar electrical characteristics. Strikingly, the drop in the *I*_*sc*_ remains fairly constant under both *T*_*m*_ conditions. On the other hand, the *V*_*oc*_ decreases substantially when *T*_*m*_ increases. Yet, at ca. 25 °C, the drop in *V*_*oc*_ appears fairly minimal. This suggests that the underlying cause for the drop in *V*_*oc*_ at ca. 40 °C is higher *T*_*m*_. The increase in *I*_*sc*_ when *T*_*m*_ increases is almost insignificant for the 3 modules (Fig. 10a–c), especially for PV Module B (Fig. b). This supports the earlier observation per the visual inspection that PV module B appears to be the most affected by optical degradation. Loss of optical transparency means that reduced amount of light gets to the solar cells during field operation. This usually manifests itself in reduced *I*_*sc*_ even when in-plane irradiance and *T*_*m*_ increases (see Fig. 10b). For Figs. a and c, the effect of *T*_*m*_ on *I*_*sc*_ is similar. There is an insignificant improvement in the *I*_*sc*_ even when *T*_*m*_ increases from 25.4 °C to 40 °C. In addition, optical degradation enhances the absorption and retention efficiency of UV light in the PV modules [24]. This leads to higher *T*_*m*_ and hence, a drop in *P*_max_ due to module hotspots [22]. Also, optical degradation induces other defects and fault modes which influence charge carrier absorption, generation, mobility, and recombination in the PV module bulk. These mechanisms influence *T*_*m*_ and hence, a drop in *V*_*oc*_ [28].

### 3.6 Temperature sensitivity

**Fig. 11:**
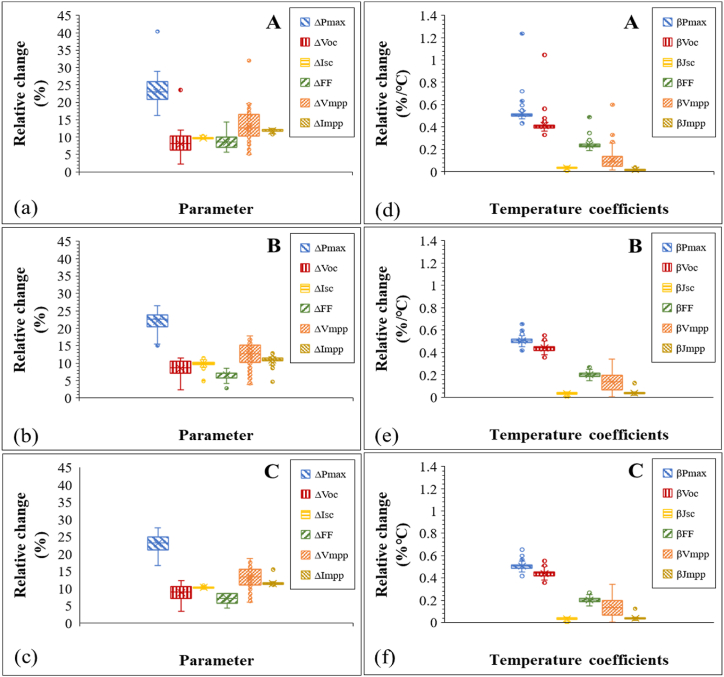
Evolution of electrical characteristics (a)–(c) and temperature coefficients (d)–(f) of the 3 field-aged PV modules, normalized to STC. The ends of the boxes are the lower and upper quartiles (interquartile range), the internal lines and *x*-marks indicate the median and mean, respectively.

Fig. 11a–c illustrate the evolution of the electrical parameters and Fig. 11d–f shows the corresponding temperature coefficients of the 3 field-aged PV modules using Box and Whisker plots. PV Module A appears to be the most degraded among the 3 field-aged PV modules, possibly due to other co-defects [5,29]. The variation in the electrical parameters, especially in the *V*_*oc*_, *FF*, and *V*_*mpp*_, is the highest, see Fig. 11a.

A close inspection of the electrical parameters shows that the relative change in *P*_max_ (Δ*P*_max_) depends more on the relative change in *V*_*oc*_ (Δ*V*_*oc*_) and *V*_*mpp*_ (Δ*V*_*mpp*_). On average, the relative percent change in *P*_max_, *V*_*oc*_, and *V*_*mpp*_ for the 3 modules are >22%, >8%, and >12%, respectively. The relative change in the fill factor (Δ*FF*) was also observed to be high, however. The higher *ΔFF* is due to carrier generation-recombination induced resistance losses and its variation with *T*_*m*_ [28]. A closer look at the evolution of the temperature coefficients of the modules (Fig. 11d–f) corresponds with the relative changes observed in the electrical parameters of the modules (Fig. 11a–c). That is, the relative change in the temperature coefficient of *P*_max_ (*β*_*Pmax*_) depends more on the relative changes in the temperature coefficients of *V*_*oc*_ (*β*_*Voc*_) and *V*_*mpp*_ (*β*_*Vmpp*_). However, the temperature coefficients predict the effects on *P*_max_ better. That is, the profile of *β*_*Pmax*_ matches that of *β*_*Voc*_ and *β*_*Vmpp*_ far better than the degree to which Δ*P*_max_ matches Δ*V*_*oc*_ and Δ*V*_*mpp*_.

**Fig. 12:**
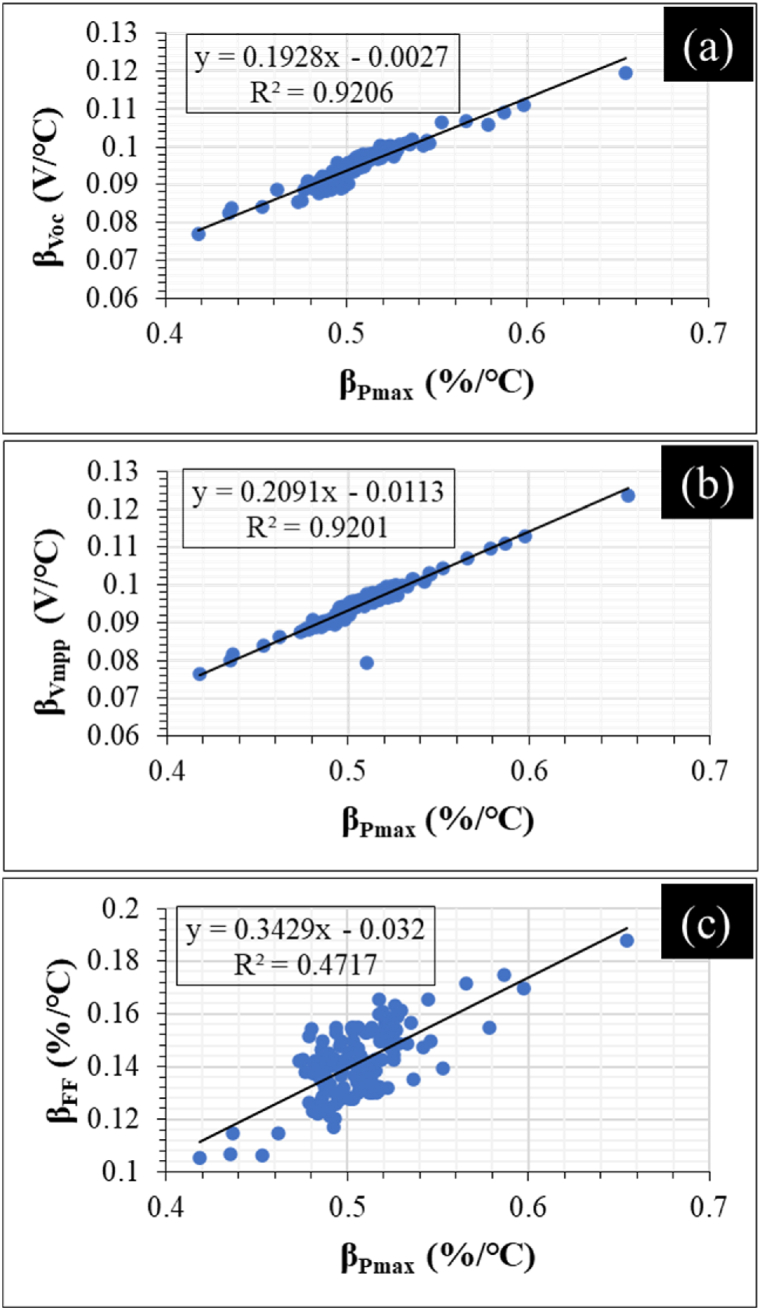
Dependence of *β*_*Pmax*_ on (a) *β*_*Voc*_, (b) *β*_*Vmpp*_, and (c) *β*_*FF*_. Strong correlation of *β*_*Voc*_ and *β*_*Vmpp*_ to *β*_*Pmax*_ (i.e., high R^2^ > 0.9) value. Weak correlation of *β*_*Jsc*_, *β*_*Jmpp*_ and *β*_*FF*_ to *β*_*Pmax*_ (i.e., low R^2^ < 0.5) value.

The relative changes in the temperature coefficients of *I*_*sc*_ (*β*_*Jsc*_) and *I*_*mpp*_ (*β*_*Jmpp*_) are far lower. However, the relative effect of *β*_*Jsc*_ and *β*_*Jmpp*_ is expected to be significant. The average *β*_*Pmax*_, *β*_*Voc*_, and *β*_*Vmpp*_ were >0.5%/°C, >0.4%/°C, and >0.1%/°C, respectively. On average, the relative change in the temperature coefficients of *FF* (*β*_*FF*_) for the 3 optically degraded PV modules, except Module A, is ca. 0.2%/°C. The variation in *β*_*Pmax*_ and *β*_*Voc*_ appear to be closely identical for all the 3 PV modules. In PV modules affected by PID, the *β*_*Pmax*_ was found to be closely identical to both *β*_*Voc*_ and *β*_*FF*_. However, for the modules affected by PID, the variation in *β*_*FF*_ was observed to be greater than 0.3%/°C [36]. To understand the degree of correlation of these electrical parameters to *P*_max_, regression plots of the temperature coefficients were carried out. The results from the regression plots of PV module C are shown in Fig. 12.

**Fig. 13:**
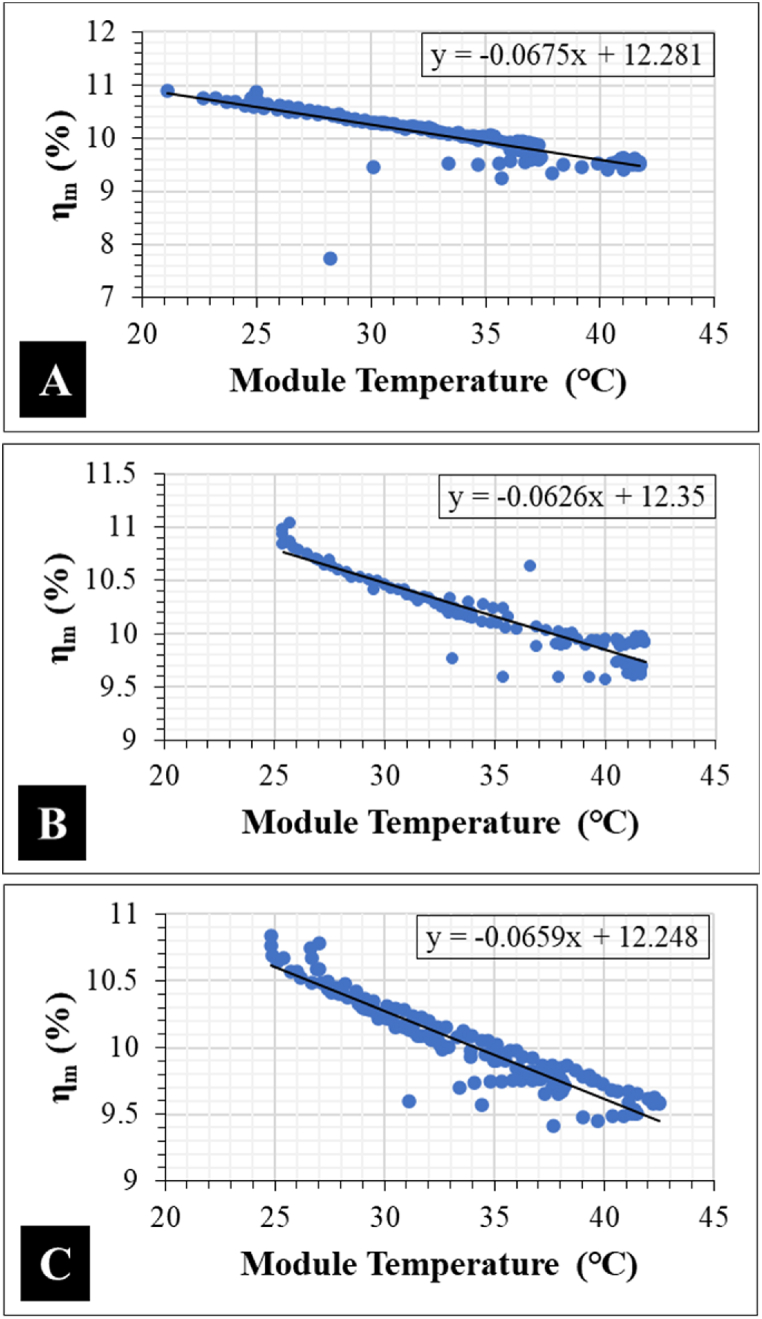
Temperature coefficient of efficiency, *β*_*ηm*_ of field-aged PV Module A, B, and C.

Fig. 12c suggests that even though *β*_*FF*_ appears to influence Δ*P*_max_, the degree of correlation is weak with R^2^ < 0.5. The R^2^ for both *β*_*Jsc*_ and *β*_*Jmpp*_ is even lower. However, *β*_*Voc*_ (Fig. 12a) and *β*_*Vmpp*_ (Fig. 12b) show a strong correlation to Δ*P*_max_ with R^2^ > 0.9 for both. These trends in the temperature sensitivity support the observed *V*_*oc*_ drop in the I–V characteristics for the optically degraded field-aged PV modules, see Fig. 10. This suggests that optical degradation in PV modules could be monitored more precisely using PV module's *β*_*Voc*_ and *β*_*Vmpp*_. However, the effect of *β*_*Voc*_ and *β*_*Vmpp*_ on *β*_*Pmax*_ also depends on other co-defects [27,36]. The regression plots of PV module efficiency (*η*_*m*_) versus *T*_*m*_ for the optically degraded PV modules are shown in Fig. 13.

In literature, the temperature coefficient of efficiency for mc-Si PV modules is 0.4%/°C [28,[31], [32], [33]]. By extrapolation, Fig. 13a–c suggests that the PV module efficiency (*η*_*m*_) for each optical degraded module at 0 °C is ca. 12%. The average temperature coefficients of the 3 optically degraded PV modules are recorded in Table 3. Our values for *β*_*Voc*_, *β*_*Jsc*_, and *β*_*Vmpp*_ agree with the values reported by King et al. [31]. In Table 3, unlike all the other parameters, the difference in the *β*_*Jmpp*_ values appears to be large for the 3 PV modules. The difference in the *β*_*Jmpp*_ is due to the degree of optical degradation in each of the PV modules. That is PV Module B (worst optically degraded module) show the highest *β*_*Jmpp*_ of 0.07%/°C. Probably, the most striking is the negative *β*_*Jmpp*_ for PV Module C.

**Table 3:** Average temperature coefficients of the 3 field-aged PV modules. These values are in good agreement with earlier reports [31,32].

PV module	Temperature coefficient (%/°C)					
	β_Voc_	β_Jsc_	β_FF_	β_ηm_	β_Vmpp_	β_Jmpp_
A	−0.4	0.04	−0.2	−0.5	−0.6	0.03
B	−0.4	0.06	−0.2	−0.5	−0.6	0.07
C	−0.4	0.04	−0.2	−0.5	−0.5	−0.01

**Fig. 14:**
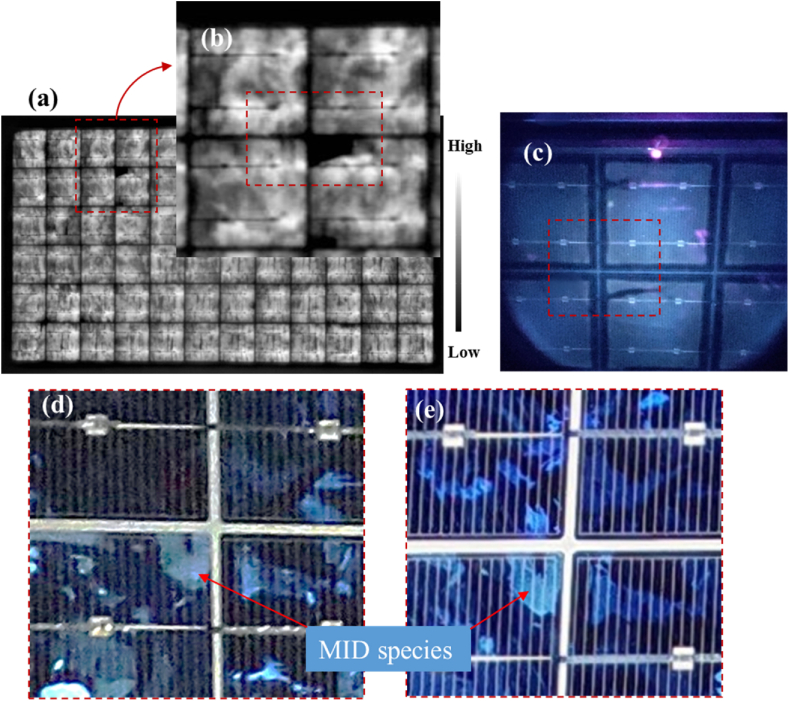
(a) EL image of PV Module C, with a corresponding (b) zoomed-in area and (c) UV-F image showing cracks (marked in red). Visual image in a (d) dark room and (e) clear sky outdoor environment showing the areas marked red in (b) and (c). These images revealed that this module is affected by other defects. Cracks can accelerate optical degradation.

Fig. 14a is an EL image of PV Module C with its corresponding zoomed in image of the marked area in Fig. 14b. Fig. 14c is an UV-F image of the corresponding area in Fig. 14b. The figure highlights other defects in addition to optical degradation. Fig. 14a–c shows microcracks and broken or severed metal grids. Fig. 14d is a visual image taken in a dark room and Fig. 14e is a visual image acquired under a clear sky outdoor environment. In both visual images, it appears that there is a sign of trapped MID species in the areas affected by cracks (as shown in Fig. 14b). However, Fig. 14d revealed more details than Fig. 14e. These defect mechanisms can influence the behaviour of current transport in the module. From the experiments, we observed that the negative *β*_*Jmpp*_ by Module C is due to reverse bias current from a critical hotspot(s) which turns the bypass diode into a heat sink. In other words, PV Module C appears to have a defective busbar due to the presence of other defects e.g., cracks, see Fig. 14. Cracks are conduit for moisture ingress, hence, can accelerate optical degradation [4,15,16]. In addition, moisture ingress can also cause PID [15,16,39]. PV modules affected by PID were found to show characteristic negative *β*_*Jmpp*_ [36]. It was observed in Sections 3, 3.3.4 that PV Module C was affected by PID. PID leads to large leakage currents [26].

So, during operation, current accumulates and forms hotspots along the defective busbar. As the *T*_*m*_ of the PV module increases, the *ΔT* of the module also increases, and the bypass diode on the string is activated which reverse bias the cells on the defective busbar. So, as the *T*_*m*_ continues to increase, the current from the cells on the defective busbars is dissipated via joule heating, according to the relation:

(2) $$P=I^{2}R=I^{2}\frac{\rho_{D}∙l_{D}}{A_{D}}$$

(3) $$\rho_{D}=R∙\frac{A_{D}}{l_{D}}$$

Where *P* is the power dissipated into heat due to defect, *I* is the current flowing through the cell, *R* and *ρ* are the resistance and resistivity in the defective cell, respectively across defect length (*l*_*D*_) and defect area (*A*_*D*_). Assuming that each defect contributes to increased resistivity in ohm-meter (Ωm), the resistivity (*ρ*_*D*_) due to defects and fault modes depends on the defect type, defect concentration, and resistance of the solar cell or module materials. Hence, the *T*_*m*_ due to dissipated energy at defective areas in the PV module depends on these factors. In defective cell or module, *R* increases significantly, and hence, the power dissipation into heat increases linearly. Therefore, *ρ*_*D*_ can be represented as a ratio of the electric field (*E*_*D*_) to the current density (*J*_*D*_) due to defects.

(4) $$\rho_{D}=\frac{E_{D}}{J_{D}}$$

Whenever *T*_*m*_ increases, *β*_*Jmpp*_ decreases and hence, *P*_max_ also decreases [27,31]. A negative *β*_*Jmpp*_ was also observed by King et al. [31].

## 4 Temperature-current dependent resistivity profiling

The resistivity (*ρ*) of a material determines how easily electricity can flow through it. In the case of PV modules, the solar cells are made of semiconducting materials with specific resistivity values [45]. When a hotspot occurs in a PV module, the *ρ* can increase. The increase in *ρ* of the solar cells due to hotspots causes a local increase in the *R* of the PV module. This increased *R* can lead to an imbalance in current flow across the module. That is, the hotspot cells may experience a decrease in current flow (due to higher *ρ*), while the cells around them experience an increase in current flow (due to lower *ρ*), refer to Eq. (4). This is because during operation, the voltage across the module remains constant, but the *ρ* in the hotspot region increases. To further explore the correlation among defect mechanisms, current flow, and *T*_*m*_, we explored the relationship between cell temperature (*T*_*c*_) and *ΔT* obtained from IR thermal images as defined by Eq. (1). This is done to understand temperature dependent resistivity in the field-aged PV modules. To this end, several IR thermal images were taken for each PV module, the *T*_*cH*_ and *T*_*cL*_ values were extracted and the *ΔT* values for each module was computed.

**Fig. 15:**
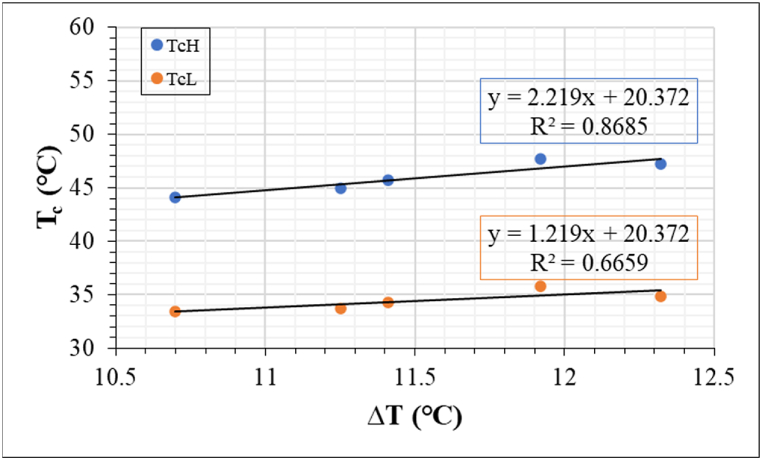
Dependence of *T*_*c*_ on difference in cell temperature (*ΔT*). The values were extracted from IR thermal images acquired under clear sky outdoor conditions for the cell with the highest temperature (*T*_*cH*_) and cell with the lowest temperature (*T*_*cL*_). In this case, *T*_*cH*_ is the hotspot cell.

Fig. 15 shows the regression plot of *T*_*c*_ versus *ΔT* for PV Module A. The correlation of the cell with the highest *T*_*c*_ (*T*_*cH*_) and the cell with the lowest *T*_*c*_ (*T*_*cL*_) to *ΔT* is shown. The correlation for *T*_*cH*_ is significantly higher (R^2^ > 0.8). This suggests that the model predicts more accurately using the *T*_*c*_ of defect areas i.e., hotspot areas. By extrapolation, Fig. 15 suggests that, when *ΔT* = 0, then the *T*_*c*_ for both *T*_*cH*_ and *T*_*cL*_ is equal. That is, when *ΔT* = 0, then the *T*_*c*_ for all the PV cells in the module is equal, hence, *T*_*c*_=*T*_*m*_ (assuming all transient mechanisms are negligible). For ‘good’ PV modules, *ΔT* is negligible and can be assumed as *ΔT* ≈ 0. As a function of efficiency, *T*_*m*_ depends largely on in-plane irradiance.

The extracted information for the graph in Fig. 15 for the 3 optically degraded PV modules is recorded in Table 4. The slopes of the cell with the lowest *T*_*c*_ (*m*_*cL*_) and highest *T*_*c*_ (*m*_*cH*_) and *T*_*m*_ of the modules are presented. Interestingly, the values for *m*_*cL*_, *m*_*cH*_, and *T*_*m*_ were observed to be constant under similar in-plane irradiance conditions.

**Table 4:** Slopes of *m*_*cL*_, *m*_*cH*_, and *T*_*m*_ of the 3 field-aged PV modules.

PV module	Slope		*T*_*m*_ (°C)
	*m*_*cL*_ (Ωm)	*m*_*cH*_ (Ωm)	
**A**	1.22	2.22	20
**B**	2.69	3.69	8.7
**C**	−0.563	0.437	40

Generally, the regression equations in Fig. 15 can be represented as

(5) $$T_{c}=m_{c}∙\Delta T+T_{m}=>\;\frac{{\partial T}_{c}}{\partial \Delta T}=m_{c}$$

where *m*_*c*_ is the slope of *T*_*cL*_ (*m*_*cL*_) or *T*_*cH*_ (*m*_*cH*_) versus *ΔT*. Fig. 15 evaluates the behaviour of current flow in the module (resistivity) as a function of temperature changes. Considering a PV module with a hotspot (due to defects), it can be assumed that during operation, the voltage across the module is constant, and that the *ρ* of the hotspot cell is higher than the *ρ* of the surrounding cells, which are in a better condition. Hence, the power dissipated into heat in the *T*_*cH*_ will be greater than the power dissipated into heat in the *T*_*cL*_ and surrounding cells due to defects induced resistivity, refer to Eq. (2). Hence, the *m*_*c*_ extracted from Fig. 15 can be used to assess current resistivity (Ωm) due to defects when temperature varies. A high *m*_*c*_ value indicates high resistivity (low conductivity), and vice versa. This is evident in Fig. 15 and Table 4 where *m*_*cH*_ is higher than *m*_*cL*_ for all the field-aged PV modules. This because *ρ*_*D*_ depends on *A*_*D*_ in defective PV cell or module, referring to Eq. (3). So, a negative *m*_*c*_ value indicates an inverse relationship between *T*_*c*_ (current flow) and *ΔT*. This means when *ΔT* increases, *T*_*c*_ decreases due to current dissipation by a critical defect such as cracks. A negative *m*_*cL*_ (−0.563 Ωm) for PV Module C supports the earlier observation where this same module showed a negative *β*_*Jmpp*_. This behaviour is characteristic of a cell with the lowest temperature in the string, since this indicates that the bypass diode is active and has been activated. On the other hand, the same module showed a positive *m*_*cH*_ (0.437 Ωm) for the hotspot, since in this case, the bypass diode might have kicked in as the reaction threshold of the bypass diode might have been exceeded.

One observation from Table 4 is that PV Module C which showed the lowest *m*_*c*_ (lowest resistivity) among the 3 modules also showed the highest *T*_*m*_ though the measurements were done under similar irradiance conditions for the 3 PV modules. This observation supports our earlier assertion that PV Module C has critical defects that inhibit current flow. Also, Module B (the worst optically degraded module) showed the highest *m*_*c*_ with corresponding lowest *T*_*m*_ of ca. 9 °C. It is noteworthy that *m*_*c*_ quantifies the bulk resistivity in the PV module. This also goes to emphasize our earlier views on the behaviour of *β*_*Jsc*_ and *β*_*Jmpp*_ due to the presence of co-defects in the module.

**Fig. 16:**
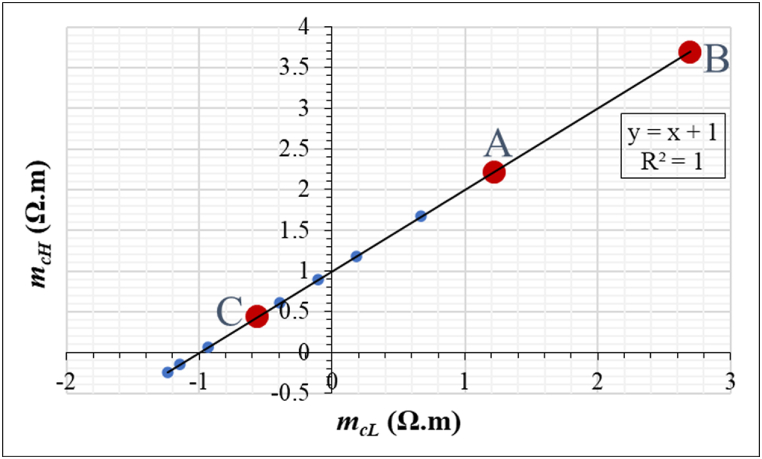
A graph of *m*_*cH*_ versus *m*_*cL*_ (extracted from IR thermal images) for 10 selected field-aged PV modules. The 10 selected PV modules are affected by different defects: optical degradation, cracks, and PID. The 3 modules affected by optical degradation are labelled A, B, and C with their position on the regression line marked in bold red dots.

Fig. 16 illustrates the relationship between *m*_*cH*_ and *m*_*cL*_ for 10 selected field-aged PV modules, including the 3 modules chosen for this investigation. Each of the 10 selected modules has been identified to be affected by at least one of the defect mechanisms: optical degradation, cracks, or PID. This is to compare the resistivity due to the *T*_*cH*_ and *T*_*cL*_ of the PV modules affected by these defect mechanisms. It appears that this technique can predict the resistivity in PV modules precisely as it shows a perfect correlation of *R*^*2*^ = 1. The 3 field-aged PV modules affected by optical degradation are highlighted in bold red dots as A, B, and C. The blue dots represent the rest of the 7 modules. The 10 PV modules are distributed in Quadrants 1, 2, and 3. The PV modules with the least and highest resistivity are located in Quadrants 1 and 3, respectively.

PV Modules A and B are located in Quadrant 1 while PV Module C is located in Quadrant 2. In Quadrant 1, Module B is located well above Module A. This suggests that Module B had the least resistance to current flow when in operation. That is, Module B appears to be the least affected by parasitic resistive current flow. On the other hand, Module C experienced the highest resistance to current flow in its bulk, which suggests that it has been affected by much more critical defects due to MID mechanisms e.g., corrosion, cracks, PID, etc. In general, it has been observed that PV modules affected by defects which are severely critical to current flow are located in Quadrants 2 and 3, hence, show negative *β*_*Jmpp*_. For instance, PV modules affected by critical cracks are located in Quadrant 3. This confirms the assertion that the severity of power loss due to optical degradation also depends on the presence of other defects.

In situations where optical degradation is caused by other defects due to MID mechanisms, the current flow in the module bulk could be influenced by the characteristics of the other co-defects such as cracks, corrosion, PID, etc. Fig. 16 suggests that the *m*_*cH*_ versus *m*_*cL*_ graph for a defect free PV module will have a slope of 1 and will be located in Quadrant 1 only. On the other hand, a module affected by uniformly distributed defects which contribute to resistance equally (rare case) will have a slope of 1 but will be located in Quadrants 1 or 3. In both of these scenarios, the *m*_*c*_ *= 1.* In the case of non-uniform defect types and distribution, *ρ*_*D*_ is likely to be non-uniform. Hence, the graph for *m*_*cH*_ versus *m*_*cL*_ will be located in Quadrants 2 or 4. The upward shift of the intercept of the graph at *m*_*cH*_ (i.e., *m*_*cH*_ > 0), indicates the influence of resistance to current flow in the module bulk due to defect induced hotspots. The model for PV cell efficiency (*η*_*c*_) or *η*_*m*_ is

(6) $$\eta_{c}=\eta_{r}\left[1-\beta \left(T_{c}-T_{r}\right)\right]$$

When *ΔT* = 0 or for ‘good’ PV module, *T*_*c*_ ≈ *T*_*m*_, then Eq. (6) becomes

(7) $$\eta_{m}=\eta_{r}\left[1-\beta \left(T_{m}-T_{r}\right)\right]$$

where *η*_*r*_ is the cell/module reference efficiency at cell/module temperature (*T*_*c*_ or *T*_*m*_) and reference temperature (*T*_*r*_) of 25 °C. The *T*_*m*_ in Eq. (7) is estimated from Fig. 15. Accounting for temperature effects, the resistivity due to defects (*ρ*_*D*_) in a PV module can be written as

(8) $$\rho_{D}=\rho_{r}\left[1-\beta \left(T_{D}-T_{r}\right)\right]$$

where *ρ*_*r*_ is the cell/module reference resistivity at the defect temperature (*T*_*D*_) and reference cell/module temperature (*T*_*r*_) of 25 °C. *β* is the temperature coefficient of the PV cell or module. For a defective PV module, *T*_*D*_ can be assumed to be *T*_*m*_. Hence, using Eq. (8), the temperature coefficient (*β*) of a PV module could be estimated from the IR thermal data of the PV module.

**Fig. 17:**
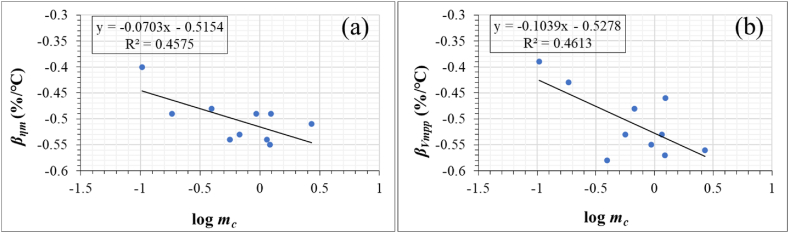
Relationship between *m*_*c*_ and (a) *β*_*ηm*_ and (b) *β*_*Vmpp*_, respectively. *m*_*c*_ is the slope of the graph of *T*_*c*_ versus *ΔT* (extracted from IR thermal images) for 10 PV modules. *m*_*cH*_ for the PV modules were used as they were more characteristic of the defects and fault modes.

Next, we explore the relationship between the information obtained from IR thermal images, *β*_*ηm*_ and *β*_*Vmpp*_ for 10 field-aged PV modules (including the 3 modules affected by optical degradation). This was done using regression plots as shown in Fig. 17.

Remember, *m*_*c*_ is the slope of *T*_*c*_ versus *ΔT*, refer to Eq. (5). The correlation between *m*_*c*_ and the temperature coefficients is weak with R^2^ < 0.5. However, by extrapolation, when log *m*_*c*_ *= 0* (i.e., when *m*_*c*_ *= 1*), *β*_*ηm*_ ≈ −0.5%/°C, refer to Fig. 17a and *β*_*Vmpp*_ ≈ −0.5%/°C, refer to Fig. 17b. From Table 3, these are the average values for these parameters at STC. It is known that the temperature sensitivity and resistivity of PV modules are highly dependent on materials properties, defect types and defects concentration, refer to Eqs. (2), (8). Hence, it appears the degradation states of the PV modules also influenced the degree of correlation as represented in Fig. 17. For instance, the regression graphs of log *m*_*c*_ versus *β*_*ηm*_ and *β*_*Vmpp*_ for PV Modules A, B, and C alone give R^2^ values greater than 0.5, but less than 0.6. The improved R^2^ value is likely due to the fact that these 3 modules are affected by a similar defect mode: optical degradation. In addition, higher polynomial regression models can be useful for understanding the relationship between the degradation-induced temperature information and the module characteristics better [46]. For instance, when a sixth order polynomial fit is used for the data in Fig. 17, the R^2^ value is greater than 0.8. Yet, the use of higher polynomial regression models can be misleading. In solar PV modeling, the model tends to be more sensitive to small changes in *G*_*I*_ when the degree of polynomial increases. The number of PV modules used may also be a factor for the low R^2^ obtained. Further studies on a larger number of defect free modules will be needed to verify and improve on the current hypothesis. Nevertheless, the use of these temperature dependent information together with other module characteristics can improve the understanding of fault diagnostics in PV plants.

## 5 Conclusion

3 field-aged PV modules that have been affected by optical degradation were investigated. The investigation made use of visual inspection, current-voltage (I–V) characterization, temperature sensitivity profiling, current resistivity profiling, ultraviolet fluorescence (UV–F), electroluminescence (EL), and infrared (IR) thermal imaging. The results show that over the 20 years (10 years outdoor operation followed by 10 years indoor storage at room temperature), the EVA encapsulants of the field-aged PV modules have undergone optical degradation: delamination, discolouration of encapsulant, metal grids oxidation and corrosion, trapped moisture/chemical species, and glass corrosion.

Visual inspection in the dark (under well controlled light exposure) as a complementary tool to the visual inspection under clear sky conditions was also used. PV modules affected by optical degradation show weak fluorescence and luminescence signal intensities. The average *ΔT* for the 3 PV modules investigated was found to be ∼10 ± 2 °C. The average degradation rate in the efficiency of the 3 PV modules was 0.8% per year. This is due to the degradation in *I*_*sc*_ due to loss of optical efficiency and the drop in *V*_*oc*_ due to high module operating temperature. It is also observed that degradation in *β*_*Pmax*_ due to optical degradation can be traced to degradation in *β*_*Voc*_ and *β*_*Vmpp*_. The average *β*_*Voc*_, *β*_*Vmpp*_, *β*_*Jsc*_, *β*_*FF*_ and *β*_*ηm*_ for the 3 modules studies were −0.4%/°C, −0.6%/°C, 0.05%/°C, −0.2%/°C, and −0.5%/°C, respectively.

A method of using the cell temperatures extracted from IR thermal imaging to estimate the degree of resistance to current flow within the PV module is also proposed. Using the temperature dependent resistivity graphs, defective modules could be identified based on the effect of defect mechanisms on current flow in the PV module. In addition, a method of extracting PV module operating temperature (*T*_*m*_) from IR thermal images is put forward. Finally, this work also demonstrates that extracting temperature coefficients directly from IR thermal data of PV modules is possible. The model for 10 field-aged PV modules with different defects and fault modes showed a weak correlation of R^2^ < 0.5. However, the model for the 3 PV modules affected by optical degradation showed R^2^ > 0.5. The proposed model in this work can be explored further and integrated into IR thermography programs in IR thermal imagers for monitoring PV plants’ reliability based on temperature sensitivity. Though these field-aged PV modules have been affected by multiple defect mechanisms, efforts were made to select these 3 modules with similar characteristics. Besides, the best way to understand degradation mechanisms in PV modules is using field-aged PV modules which are exposed to multiple environmental stressors, and hence, suffer from multiple defect and fault mechanisms. This work has the potential of improving upon the existing knowledge on faults diagnostics in PV plants.

## Author contribution statement

Oscar Kwame Segbefia: conceived and designed the experiments; performed the experiments; analyzed and interpreted the data; wrote the paper.

## Data availability statement

Data will be made available on request.

## Additional information

No additional information is available for this paper.

## Declaration of competing interest

The authors declare that they have no known competing financial interests or personal relationships that could have appeared to influence the work reported in this paper.
